# Functional Laparoscopic Roux-en-Y Gastric Bypass with Fundectomy and Gastric Remnant Exploration (LRYGBfse)—a Video Vignette

**DOI:** 10.1007/s11695-021-05298-w

**Published:** 2021-02-22

**Authors:** Giovanni Lesti, Marco Antonio Zappa, Francesco Lesti, Davide Bona, Alberto Aiolfi

**Affiliations:** 1 Department of General Surgery, Fondazione Salus Clinica Di Lorenzo, Via Vittorio Veneto 37, Avezzano, AQ Italy; 2 Department of General and Emergency Surgery, Ospedale Fatebenefratelli Sacra Famiglia, University of Milan, Milan, Italy; 3 Department of Biomedical Science for Health, Division of General Surgery, Istitituto Clinico Sant’Ambrogio, University of Milan, Milan, Italy

**Keywords:** Laparoscopic functional gastric bypass, Bariatric surgery, Gastric bypass, Video vignette

## Abstract

**Background:**

The laparoscopic Roux en-Y gastric bypass (LRYGB) is performed worldwide and is considered by many the gold standard treatment for morbid obesity. However, the difficult access to the gastric remnant and duodenum represents intrinsic limitations. The functional laparoscopic gastric bypass with fundectomy and gastric remnant exploration (LRYGBfse) is a new technique described in attempt to overcome the limitations of the LRYGB. The purpose of this video was to demonstrate the LRYGBfse in a 48-year-old man with type II diabetes and hypertension.

**Methods:**

An intraoperative video has been anonymized and edited to demonstrate the feasibility of LRYGBfse.

**Results:**

The operation started with the opening of the gastrocolic ligament. Staying close to the gastric wall, the stomach is prepared up to the angle of His. After the placement of a 36-Fr orogastric probe, gastric fundectomy is completed in order to create a 30cc gastric pouch. A polytetrafluoroethylene banding (ePTFE) is placed at the gastro-gastric communication, 7cm below the cardia, and gently closed after bougie retraction. The bypass is completed by the creation of an antecolic Roux-en-Y 150cm alimentary and 150cm biliopancreatic limb.

**Conclusion:**

The LRYGBfse is a feasible and safe technique. The possibility to endoscopically explore the excluded stomach with an easy access to the Vater’s papilla is a major advantage. Further studies are warranted to deeply explore and compare outcomes with the standard LRYGB.

**Supplementary Information:**

The online version contains supplementary material available at 10.1007/s11695-021-05298-w.

## Introduction

The laparoscopic Roux en-Y gastric bypass (LRYGB) is performed worldwide and is considered by many the gold standard treatment because of its excellent results in term of weight loss, comorbid resolution, and quality of life improvement [1, 2]. However, the challenging diagnosis and treatment of developing diseases in the gastric remnant, duodenum, and common bile duct are limits [3]. The functional laparoscopic gastric bypass with fundectomy and gastric remnant exploration (LRYGBfse) is a relatively new technique introduced in attempt to overcome the limitations of the LRYGB with promising preliminary results [4].

## Purpose

The purpose of this video was to demonstrate the fashioning of a LRYGBfse in a 48-year-old morbidly obese man (weight 147 kg; BMI 42.8 kg/m^2^) with hypertension and type II diabetes.

## Methods

An intraoperative video has been edited to demonstrate the feasibility of the LRYGBfse. Written informed consent was obtained from the patient.

## Results

The operation started with the opening of the gastrocolic ligament. Staying close to the gastric wall, the stomach that is prepared up to the angle of His and care is taken to seal the short vessels and to free any posterior adhesions. The left pillar dissection is performed meticulously and its fibers need to be exposed. After the placement of a 36-Fr orogastric probe, the gastric fundectomy is completed with sequential Endo GIA^TM^ linear stapler firings (Medtronic, Minneapolis, MN, USA) in order to create a 30cc gastric pouch. A polytetrafluoroethylene banding (ePTFE) is placed at the gastro-gastric communication (7cm below the cardia) and gently closed after bougie retraction. The bypass is completed by the creation of an antecolic Roux-en-Y 150cm alimentary and 150cm biliopancreatic limb. The common channel is measured about 300cm. In case the common channel is shorter than 300cm, the alimentary limb is shortened. Linear side-to-side gastrojejunal and jejuno-ileal anastomosis (30-mm Endo GIA™) are fashioned. The Petersen’s defect is closed with absorbable sutures.

## Discussion

The LRYGBfse has been described in attempt to overcome the limitations of the LRYGB with encouraging preliminary results [3, 4]. The creation of a gastro-gastric virtual communication is the result of a specific and well-calibrated surgical procedure. The ePTFE band gently closes the gastro-gastric communication, and the bolus is diverted through the alimentary limb with duodenal and jejunal functional exclusion. As documented by the postoperative gastrographin swallow study, the radiopaque bolus is rapidly diverted through the gastrojejunal anastomosis and the alimentary limb. The prompt arrival of bolus in the foregut and hindgut results in an increased glucagon-like peptide 1 (GLP-1) and peptide YY (PYY) secretion [5].

The resection of the gastric fundus is performed for hormonal, physiological, and technical reasons with a significantly reduced secretion of ghrelin and consequent effect on glucose homeostasis and appetite [6]. These findings suggest that the fundus resection with concomitant ghrelin suppression could be an effective adjunct in the treatment of type II diabetes [7].

**Fig. 1:**
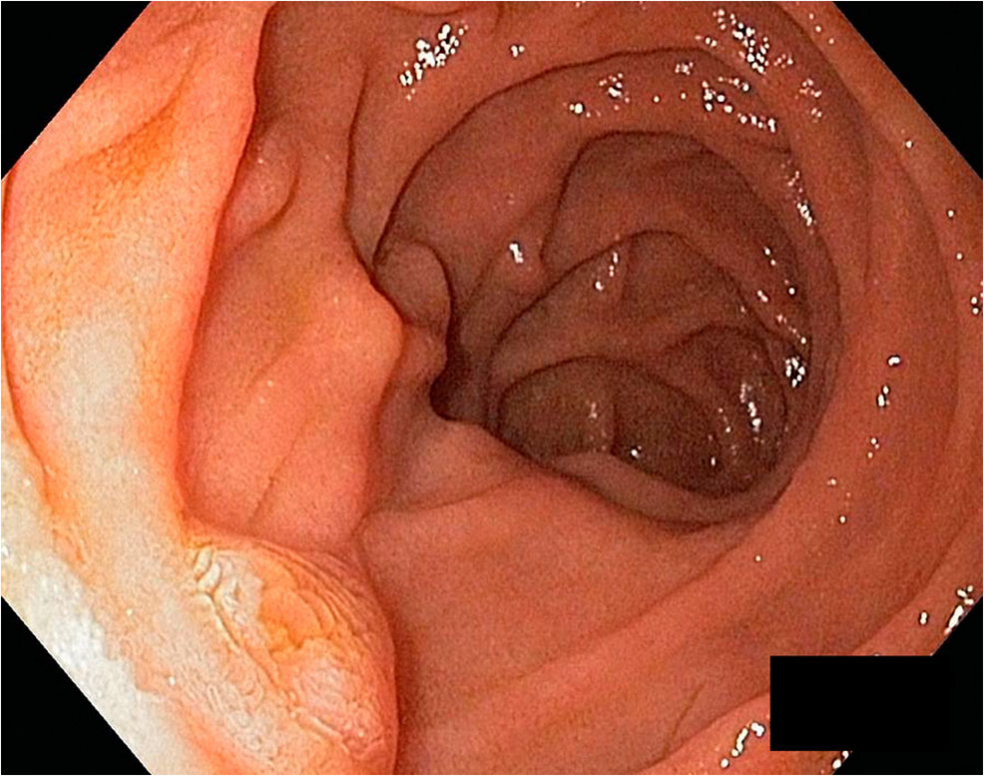
The duodenum and the Vater’s papilla may be reached and explored with 9-mm and 13-mm endoscopes for diagnostic and therapeutic purposes

Notably, the passage of 9-mm and 13-mm endoscopes for diagnostic and therapeutic purposes is allowed towards the gastro-gastric communication by gentle pushing (Fig. 1) [4]. Systematic endoscopic exploration of the gastric remnant and duodenum are performed yearly during follow-up. Endoscopic retrograde cholangiopancreatography with sphincterotomy was necessary in two patients for symptomatic choledocholithiasis, and another patient was diagnosed with prepyloric cancer 15 months after the index procedure. The feasible exploration of the remnant remains a critical issue considering the increasing number of LRYGB performed each year worldwide in a population of young patients that will probably need a screening evaluation in the future [8, 9].

## Conclusion

The LRYGBfse is a new feasible and safe technique. The possibility to endoscopically explore the excluded stomach with an easy access to the Vater’s papilla is a major advantage. Further studies are warranted to deeply explore and compare outcomes with the standard LRYGB.

## Supplementary Information

Below is the link to the electronic supplementary material.

ESM 1 (MP4 373047 kb)
